# CRISPR-mediated knockout of *cardinal* and *cinnabar* eye pigmentation genes in the western tarnished plant bug

**DOI:** 10.1038/s41598-022-08908-4

**Published:** 2022-03-22

**Authors:** Chan C. Heu, Roni J. Gross, Kevin P. Le, Dannialle M. LeRoy, Baochan Fan, J. Joe Hull, Colin S. Brent, Jeffrey A. Fabrick

**Affiliations:** USDA ARS, U.S. Arid Land Agricultural Research Center, Maricopa, AZ 85138 USA

**Keywords:** RNAi, Functional genomics, Agricultural genetics, Genetic markers

## Abstract

The western tarnished plant bug, *Lygus hesperus*, is a key hemipteran pest of numerous agricultural, horticultural, and industrial crops in the western United States and Mexico. A lack of genetic tools in *L. hesperus* hinders progress in functional genomics and in developing innovative pest control methods such as gene drive. Here, using RNA interference (RNAi) against *cardinal* (*LhCd*), *cinnabar* (*LhCn*), and *white* (*LhW*), we showed that knockdown of *LhW* was lethal to developing embryos, while knockdown of *LhCd* or *LhCn* produced bright red eye phenotypes, in contrast to wild-type brown eyes. We further used CRISPR/Cas9 (clustered regularly interspaced palindromic repeats/CRISPR-associated) genome editing to generate germline knockouts of both *LhCd* (Card) and *LhCn* (Cinn), producing separate strains of *L. hesperus* characterized by mutant eye phenotypes. Although the *cardinal* knockout strain Card exhibited a gradual darkening of the eyes to brown typical of the wild-type line later in nymphal development, we observed bright red eyes throughout all life stages in the *cinnabar* knockout strain Cinn, making it a viable marker for tracking gene editing in *L. hesperus*. These results provide evidence that CRISPR/Cas9 gene editing functions in *L. hesperus* and that eye pigmentation genes are useful for tracking the successful genetic manipulation of this insect.

## Introduction

The western tarnished plant bug, *Lygus hesperus* Knight (Hemiptera: Miridae) is a major pest of cotton and other crops throughout the western United States and other parts of North America^1–3^. Although an integrated pest management program has been implemented against *L. hesperus* in Arizona^3^, its success is dependent on the continued effectiveness of only a few insecticides which have been widely used for many years^4^. With the rapid evolution of insecticide resistance observed in the closely related *Lygus lineolaris*^5–7^, new tactics are needed to maintain control over members of this genus.

Among contemporary strategies for controlling arthropod pest species, homing-based gene drives are currently being developed for management of agricultural pests as well as those that vector human disease^8–13^. Clustered regularly interspaced short palindromic repeats and CRISPR-associated protein 9 (CRISPR/Cas9) gene drive systems have been developed and shown to effectively drive deleterious genes into laboratory insects, resulting in population crashes^11,13,14^. CRISPR-based gene drive systems developed in the laboratory include homing, split homing, translocation, X-shredder, killer-rescue, cleave-and-rescue, and TARE (reviewed in^15^).

CRISPR/Cas9 gene editing is also widely used to ascertain gene function due to its efficiency and specificity in inducing mutations by cleavage and impairment of the genomic target sequences in model and non-model organisms^16,17^. In insects, genes that control eye pigmentation are frequently targeted because many of the induced mutations produce striking visible changes that facilitate screening for knockout efficacy. A frequent target is White, an ABC transporter that functions in transporting pigment precursor into pigment granules^18^. For example, CRISPR editing of *white* produced white-eyed adults in both *Helicoverpa armigera*^19^ and *Bactrocera dorsalis*^20^, although homozygous mutations were lethal in the former and unexpectedly resulted in the loss of black head spots in the latter. In hemipterans, CRISPR-mediated null mutation of *white* results in white eyes in nymphs and bright red eyes in adults of *Bemisia tabaci*^21^ and lighter red eyes and white ocelli in *Nilaparvata lugens*^22^.

**Figure 1:**
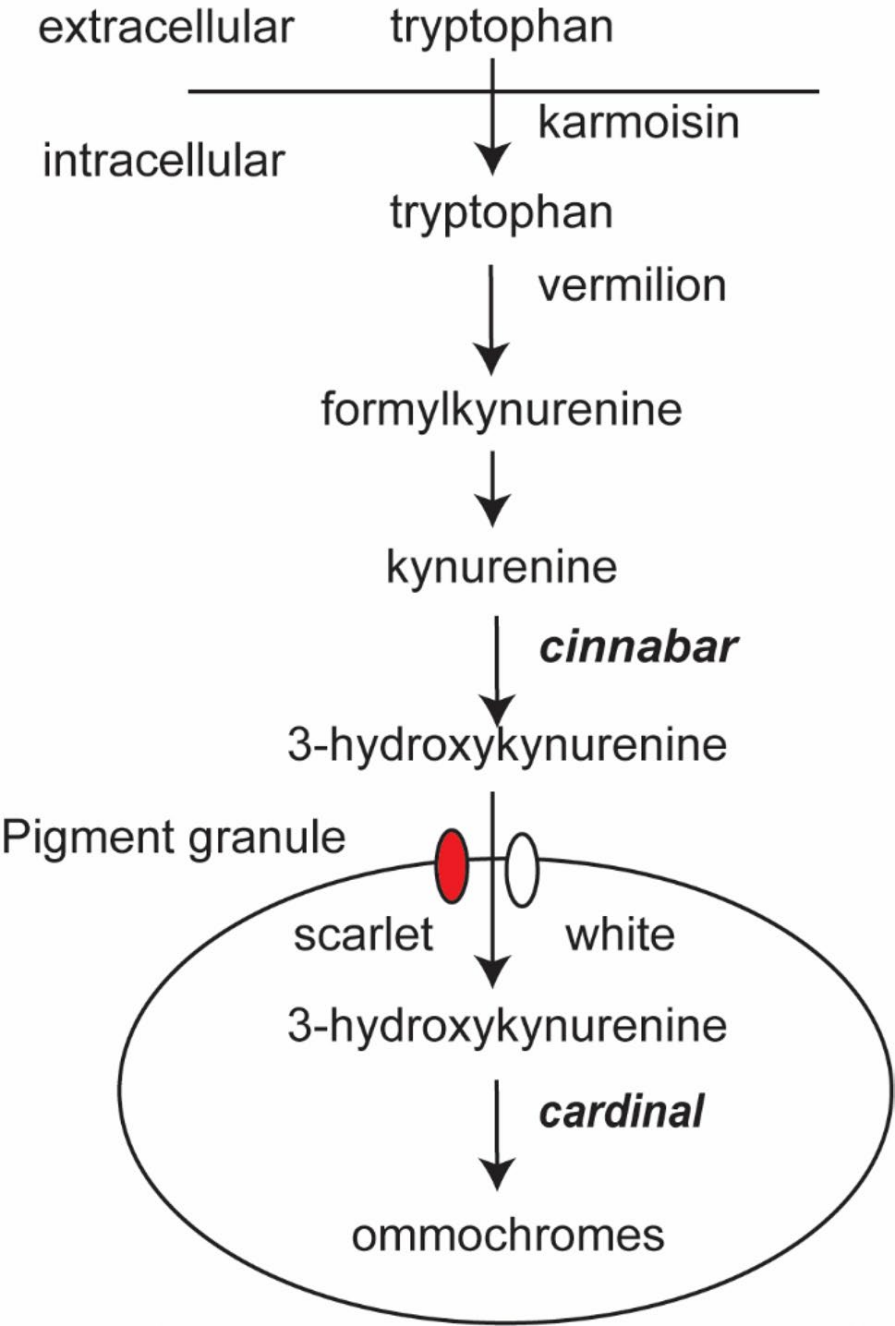
The insect ommochrome eye pigmentation pathway. Genes involved are *karmoisin*, *vermilion*, *cinnabar*, *scarlet*, *white*, and *cardinal*. Genes targeted for gene function in *L. hesperus* are italicized.

Insect eye pigments are primarily from the guanine-derived pteridines and/or the tryptophan-derived ommochromes. Genes involved in the ommochrome pathway of pigment transport and formation are well studied in several model species, including *Drosophila melanogaster*, *Bombyx mori* and *Tribolium castaneum*^23–25^ and typically follow the schematic shown in Fig. 1. Null mutations in the enzymes involved in the step-by-step process of converting tryptophan into ommochromes (Vermilion, Cinnabar, and Cardinal) within this pathway often produce distinct eye color phenotypes and serve as visible markers for detecting successful gene manipulation. In *Helicoverpa zea*, mutant yellow eyes were observed after CRISPR-mediated knockout of *vermilion*, tryptophan 2,3-dioxygenase^26^. Knockouts of kynurenine monooxygenase (*cinnabar*) in *Plutella xylostella* manifested in yellow or red eyes depending on the mutation, while knockout of the haem peroxidase gene, *cardinal,* produced yellow eyes that gradually changed to red^27^. In *N. lugens*, knockout of *cinnabar* generated a red eye phenotype^22^. Such studies demonstrate that genes in the ommochrome pathway like *vermilion*, *cinnabar*, and *cardinal* can serve as targets that give discernable phenotypes for tracking stable germ line gene edits across multiple generations.

Current knowledge of eye pigmentation in Hemiptera is limited to just a few species and has been produced primarily through genetic manipulation of *white*, *cinnabar*, and *cardinal*^20,24,28–30^. In *L. lineolaris*, a mutant red eye phenotype occurs naturally under field and laboratory conditions^6,31,32^, although the genetic basis of this phenotype is unknown. To reproduce such mutants in *L. hesperus*, we previously used RNAi to knockdown genes in the ommochrome pathway. In that study, late 5th instar nymphs were injected with dsRNA and eye color development was tracked through adult maturation seven days post-eclosion^28^. However, RNAi only produced red pigment along the margins of the eyes which otherwise looked wild-type. We concluded that the incompleteness of the transformation to a fully red eye was the result of the transient knockdown of the genes and the accrual of pigments during nymphal development^28^. Thus, an induced null mutation in genes involved in either the synthesis or transport of ommochromes may lead to a more pronounced adult phenotype.

Here, we aim to demonstrate that CRISPR/Cas9 can be applied to *L. hesperus* by targeting genes within the ommochrome pathway. We show that CRISPR/Cas9-induced mutations in *cardinal* (*LhCd*) and *cinnabar* (*LhCn*) are heritable and that stable lines (named Card and Cinn, respectively) with obvious mutant eye color phenotypes can be established for both genes. Although the Card strain showed pronounced red eyes early in development, coloration eventually reverted to wild-type in late nymphs and adults, suggesting that other genes are involved in driving eye pigmentation in the later stages of *L. hesperus* development. In contrast, we observed stable red eyes in the Cinn strain, suggesting that *LhCn* is critical for eye pigmentation throughout development and can potentially be used to track transformation and gene drive experiments that could ultimately lead to alternative *L. hesperus* control measures.

## Results

### Knockdown of *cardinal*, *cinnabar*, and *white* in embryos

**Figure 2:**
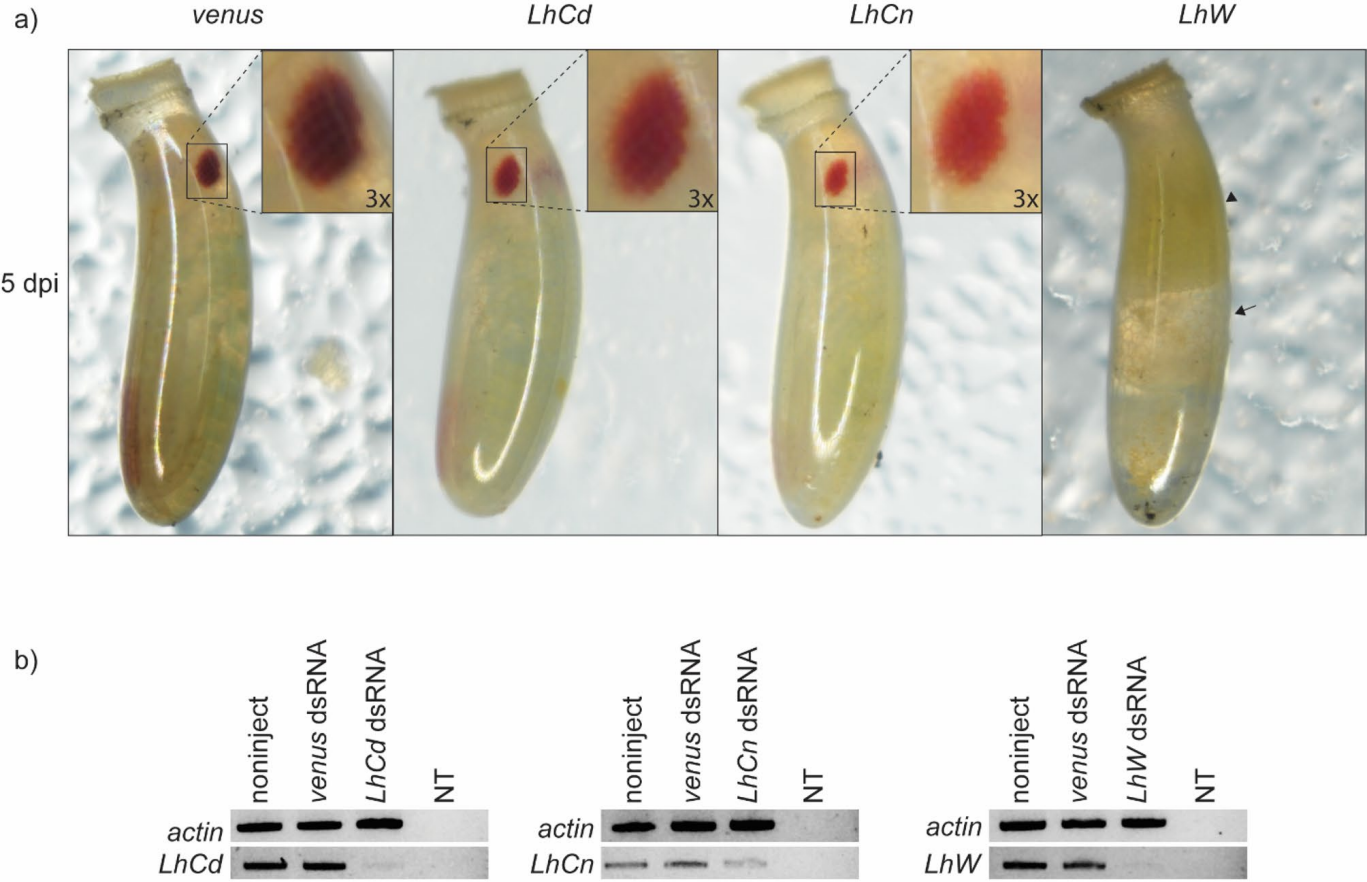
RNA interference knockdown of embryonic eye pigment genes. (**a**) *Lygus hesperus* embryos at five days post-injection (dpi) with dsRNA for *venus*, *LhCd*, *LhCn*, or *LhW*. Embryos injected with dsRNA corresponding to *LhW* were lethal and resulted in no accumulation of eye pigmentation. Inset shows 3 × magnification of the eyes. Arrowhead and arrow indicate the undeveloped and unorganized but developed parts of the *LhW* dsRNA injected egg, respectively. (**b**) Semi-quantification of knockdown in *LhCd*, *LhCn*, and *LhW* transcripts. Gel images of transcript knockdown are representative of four technical replicates and three biological replicates. No template (NT).

Using embryonic RNAi, we were able to pre-determine the effects of CRISPR/Cas9-mediated mutations on eye color. In our previous work, RNAi knockdown of *LhCd* or *LhCn* in late 5th instars resulted in mature adult eyes that had a bright red band along the medial margins, whereas *white* (*LhW*) knockdown led to a high proportion of incomplete adult molts and a commensurate increase in mortality^28^. Here, injecting dsRNAs targeting either *LhCd* or *LhCn* into ~ 1 h old *L. hesperus* eggs produced in embryos medium red or bright red eyes, respectively (Fig. 2a). These phenotypes were readily apparent 5 days post-injection and differed from brown wild-type eyes. In total, the observed phenotypes were present in 21/120 eggs injected with *LhCd* dsRNA and 28/120 eggs for the *LhCn* dsRNA treated group. Among the remaining eggs injected with either *LhCd* or *LhCn* dsRNA, 30–50% were either dead or had wild-type eyes.

Like the post-eclosion lethality previously observed following *LhW* knockdown in 5th instar nymphs^28^, embryonic injection of *LhW* dsRNA resulted in nonviable eggs characterized by incomplete and/or unorganized embryonic development at 5 days post-injection (Fig. 2a). This phenotype, which is distinguishable from the complete lack of development observed when physical trauma (*i.e.,* injection) induces egg mortality, was observed in 53/120 eggs (Fig. 2a). Knockdown of *LhCd*, *LhCn*, and *LhW* transcripts was confirmed by semi-quantitative PCR (Fig. 2b, Supplementary Fig. S1).

### CRISPR/Cas9-mediated knockout of *LhCd *or *LhCn*

Given the phenotypes generated in the embryonic RNAi experiments, we next sought to assess the viability of embryonic CRISPR/Cas9 knockouts singly targeting *LhCd* and *LhCn.* For both transcripts, the sequences corresponding to the 20-nucleotide guide and the protospacer adjacent motif were searched via BLASTn against *L. hesperus* publicly available data (NCBI organism limit—*L. hesperus*; taxid 30085). No matches were found that would suggest potential off-target effects (Supplementary Table S1).

The CRISPR/Cas9 knockouts were conducted via injections in two independent experiments. Overall, we achieved a hatch rate between 8.8 and 27.5% for injected individuals compared to 86.3–100% for the noninjected control group (Supplementary Table S2). The survival of the instar nymphs to adults ranged from 22.7 to 71.4% for all Cas9-ribonucleoprotein complex (RNP) injected groups (Supplementary Table S2). Of these RNP-injected adults, 40–100% showed mutant eye phenotypes (Supplementary Table S2).

**Figure 3:**
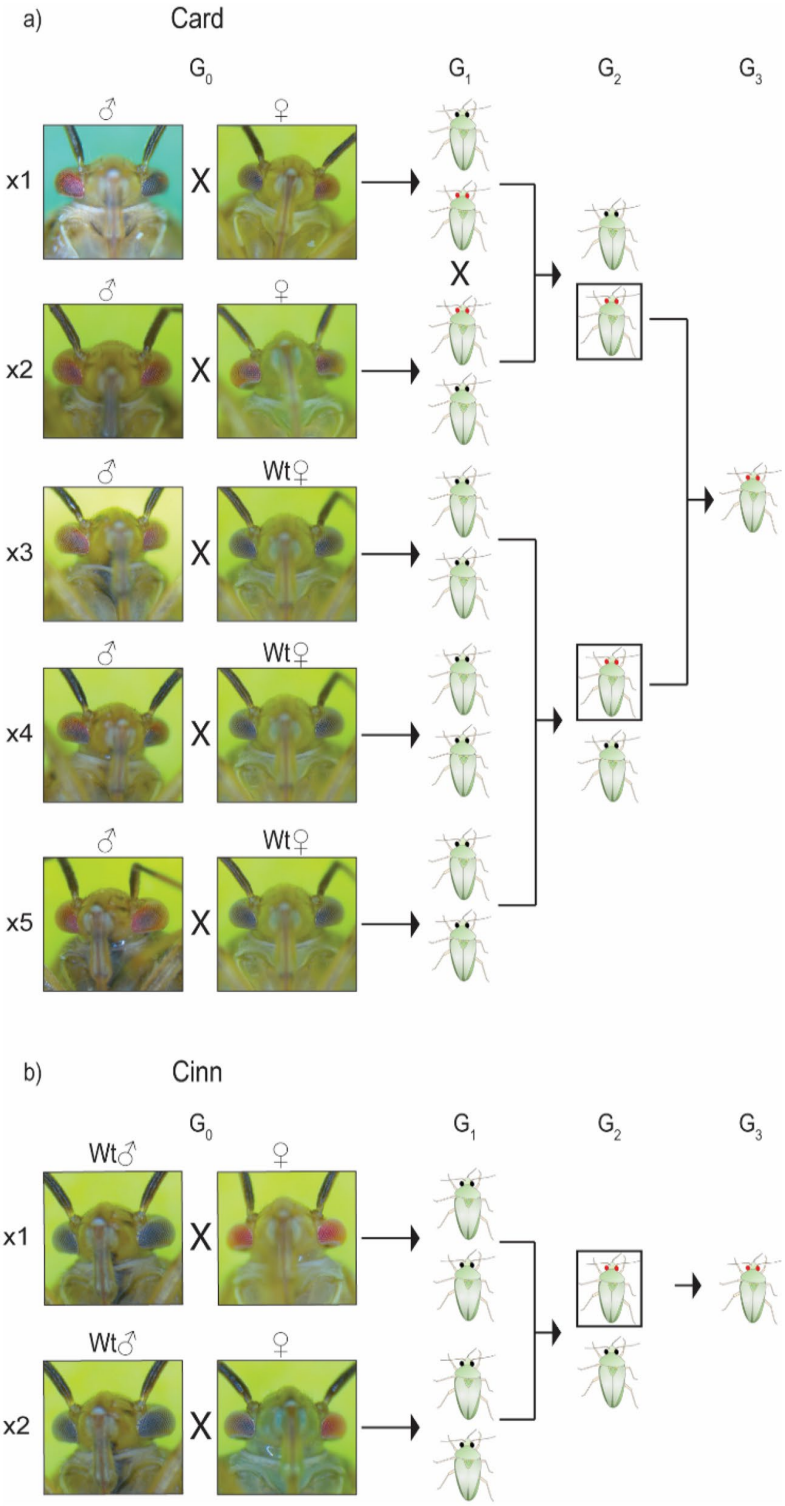
Crossing schemes used to generate CRISPR/Cas9 *Lygus hesperus* mutant eye pigmentation strains. (**a**) The Card strain was initiated from six G_0_ survivors (two females and four males). Both mutant G_0_ females were crossed with a mutant male (Card × 1 and × 2) and the remaining mutant males were crossed with four wild-type females (Card × 3–5). G_1_ offspring of Card × 1 and × 2 were inbred to generate G_2_. G_1_ heterozygous offspring from Card × 3–5 were inbred to generate a mix of wild-type and red-eye G_2_ progeny. The selected G_2_ (boxed) with red eyes from all Card crosses were pooled to establish the Card colony. Cross numbers are indicated by an “x” and the respective number. (**b**) Two G_0_ female from Cinn were crossed with wild-type males generating heterozygous G_1_ progeny that were subsequently inbred to produce G_2_. Red-eye mutants (boxed) in G_2_ were crossed and perpetuated to establish the Cinn colony. *L. hesperus* models with black or red eyes represent wild type or mutant, respectively, in (**a**) and (**b**).

To generate *L. hesperus* strains with the mutant eye phenotypes, we crossed surviving G_0_ adults from both the *LhCd-* and *LhCn*-injected lines from experiment 1 according to the scheme depicted in Fig. 3. We estimate that the efficiency of CRISPR/Cas9 gene-editing of the germline was 86% (± 9.6%), as determined by averaging the efficiency of all samples from the Card and Cinn strains (Supplementary Methods and Table S3). Individuals from the Card strain showed a gradual darkening of the eye beginning at the 3rd instar that continued with subsequent molts and during adult maturation (Fig. 5a). Eye coloration in fully mature adult Card females closely resembled those from wild-type, while Card males displayed red eye phenotypes that were brighter than those found in the wild-type strain (Fig. 5b). In contrast, the eye phenotype across all stages of Cinn strain development was characterized by complete bright red pigmentation regardless of sex and stage (Fig. 5).

### Target site mutations in *LhCd *and *LhCn* mutant strains

**Figure 4:**
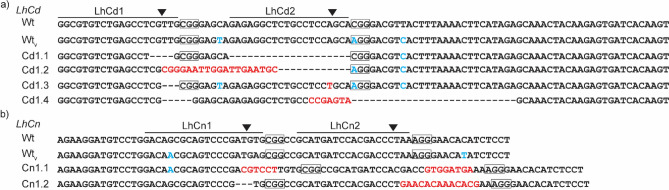
Intergenerational *LhCd* and *LhCn* mutations in *Lygus hesperus* CRISPR/Cas9 mutant strains. Multiple sequence DNA alignments of intergenerational *LhCd* (**a**) and *LhCn* (**b**) mutations with the corresponding wild-type (Wt) sequences. Aligned regions of *LhCd* and *LhCn* only include sequences near the sgRNA target sites (LhCd1-2 and LhCn1-2). A variant allele within the wild-type sequences (Wt_v_) is shown. Horizontal lines indicate the sgRNA sequences, boxes indicate the PAM sequence trinucleotides, arrowheads indicate the predicted Cas9 cut sites, blue text indicates allele variants, red text denotes substitutions or insertions, and (–) corresponds to a sequence deletion.

Both gDNA and cDNA corresponding to the gRNA target sites in G_0_-G_3_ individuals from the Card and Cinn strains showed mutations (Table 1). For *LhCd*, we found a total of 43 mutations with 20 corresponding to LhCd1 sgRNA1 and 23 for LhCd2 sgRNA2 (Table 1, Fig. 4). For *LhCn*, we found 12 and 13 mutations corresponding to LhCn1 sgRNA1 and LhCn2 sgRNA2, respectively (Table 1, Fig. 4). Of these mutations, 25 unique combinations were found for *LhCd* and 14 for *LhCn*. Names of the mutations are based on the unique combination of mutations that were found within each individual (Table 1). With the only exception of mutation Cd1.7, in which *LhCd* showed a wild-type allele at LhCd1 target site, all targeted sites displayed more than one mutation. Four *LhCd* mutations (Cd1.1-Cd1.4) and two *LhCn* mutations (Cn1.1 and Cn1.2) were found to occur in gDNA and/or cDNA across generations (Table 1). Cd1.1 was the most common *LhCd* mutation, as it was found in a single G_0_ individual and in two G_3_ individuals from the Card strain (Table 1, Fig. 4). For the Cinn strain, two individuals, including one G_0_ and one G_2,_ both harbored the Cn1.1 mutation (Table 1, Fig. 4). There were 10 mutations in Card and 5 mutations in Cinn that were in-frame, whereas 14 and 9 mutations in Card and Cinn, respectively generated premature stop codons. Overall, the combination of mutations at the sgRNA1 and 2 target sites resulted in mutant eye color phenotypes regardless of the in-frame effect.

**Table 1:** CRISPR/Cas9 induced *LhCd* and *LhCn* mutations in *Lygus hesperus*.

Gene	Name^a^	Mutation corresponding to sgRNA target sites		Type^e^	gDNA		cDNA	
		sgRNA 1^bc^	sgRNA 2^bd^		G_n_	# of samples (# of clones)	G_n_	# of samples (# of clones)
***LhCd***								
	Cd1.1	664_667del	677_696del	ps	0, 3	1 (1), 2 (11)	nd^f^	nd
	Cd1.2	666_697delinsCGGGAATTGGATTGAATGC	*^g^	InF	nd	nd	2, 3	1 (1), 1 (4)
	Cd1.3	666_668del	693A > T	InF	1	1 (5)	1, 2	1 (1), 2 (4)
	Cd1.4	666_670del	690_723delinsCCGAGTA	ps	3	1 (6)	2	5 (9)
	Cd1.5	666_669del	683_697delinsGCACGCAACATGGCTGGCAA	ps	1	1 (5)	1	2 (5)
	Cd1.6	664_666del	690_692delinsCTCTGT	InF	0	1 (1)	nd^g^	nd
	Cd1.7	wt^h^	692del	ps	0	1 (1)	nd	nd
	Cd1.8	649_665delins^j^	680_692delinsG	nd	0	1 (1)	nd	nd
	Cd1.9	666_692delinsGG	*	ps	0	1 (1)	nd	nd
	Cd1.10	666_670del	688_693del	ps	0	1 (1)	nd	nd
	Cd1.11	666_670del	689_695del	ps	0	1 (1)	nd	nd
	Cd1.12	649_665delinsTGGGTTTTCCCGAAGGC	670_693delinsCTCACGAGCTCCCTCCTACCCCTT	InF	0	1 (1)	nd	nd
	Cd1.13	666_670del	690_695del	ps	0	1 (1)	nd	nd
	Cd1.14	666_694delinsGACGCAAGGACG	*	ps	2	1 (6)	nd	nd
	Cd1.15	664_669delinsTTTG	693_694insA	ps	nd	nd	1	1 (2)
	Cd1.16	659_668delinsTCGTTAGTGTTG	684_697delinsAC	InF	nd	nd	1	1 (2)
	Cd1.17	666_693delinsTTGCGGGAATTGGATTGAAT	697del	InF	nd	nd	1	2 (2)
	Cd1.18	664_668delinsTG	690_697delinsGGAGCCTGCCGGAGCAA	InF	nd	nd	2	1 (1)
	Cd1.19	666_671del	693_697delinsTGCTCTGCAA	ps	nd	nd	2	1 (3)
	Cd1.20	664_668delinsG	675_694del	InF	nd	nd	3	1 (4)
	Cd1.21	664_668delinsTG	675_694del	ps	nd	nd	3	1 (2)
	Cd1.22	666_697delinsGACGCAAGGACGCAA	*	ps	nd	nd	2	1 (1)
	Cd2.1	665_666insGAGT	689_695del	InF	nd	nd	1, 1, 1	1 (2), 1 (5), 1 (1)
	Cd2.2	660_666del	680_694del	InF	nd	nd	1	1 (5)
	Cd2.3	664_665del	691_693delinsAGGCTCTGCCGGCTCT	ps	nd	nd	1	1 (2)
***LhCn***								
	Cn1.1	220_221insCGTCCT	245_246delinsGTGGATGA	ps	0, 2	1 (5), 1 (5)	2	1 (2)
	Cn1.2	220_222del	246_247insGAACACAAACACG	ps	2, 3	4 (16), 3 (18)	2, 3	4 (16), 2 (9)
	Cn1.3	220_221del	244_246delinsG	ps	0	1 (1)	nd	nd
	Cn1.4	221_223del	244_247delinsG	InF	0	1 (1)	nd	nd
	Cn1.5	223 T > A	246_247insGAACATAACGGGAACATAAT	ps	0	1 (2)	nd	nd
	Cn1.6	223_224del	246del	InF	0	1 (2)	nd	nd
	Cn2.1	219_227delinsATGTTTTC	230_342del	InF	nd	nd	1	1 (3)
	Cn2.2	182_228delinsAA	234_246delinsACATCTCCTCTCTCTCA	InF	nd	nd	1, 1	1 (1), 1 (5)
	Cn2.3	221_222delinsCA	243_248del	InF	nd	nd	1	1 (4)
	Cn2.4	221_222del	243_248delinsAA	ps	nd	nd	1	1 (1)
	Cn2.5	220_222del	245_247delinsTGAACACAAACACG	ps	nd	nd	1	1 (1)
	Cn2.6	220_222del	246del	ps	nd	nd	1	1 (5)
	Cn2.7	221_222insT	247_248insTCGA	ps	nd	nd	1	1 (5)
	Cn2.8	219del	246_251delinsGCGGGAGCGGG	ps	nd	nd	1	1 (1)

^a^Mutation name defines the combination of alleles at sgRNA1 and sgRNA2 target sites in an individual as a single mutation.

^b^Mutation nomenclature, showing the nucleic acid sequence changes in *LhCd* and *LhCn*, are based on the recommendations by the Human Genome Variation Society (http://www.hgvs.org/), with modification to omit the “prefix.” Mutations within 10 nucleotides were considered one mutation event.

^c^Mutations for sgRNA1 corresponding to either *LhCd* or *LhCn.*

^d^Mutations for sgRNA2 corresponding to either *LhCd* or *LhCn.*

^e^Type of mutation (ps, premature stop; InF, in-frame mutation, nd, mutation type cannot be determined because the putative splice site was affected).

^f^nd = not determined.

^*g^Mutation corresponding to sgRNA 1 that also spans the sgRNA 2 target site.

^h^wt = wild-type allele.

^i^Sequence corresponding to the delins was too long for the respective table column: 649_665delinsTAAATTGTACAATTTATTGGCCAACTATTTCTAAAGACGGTTATCATGACATAAATACCTAATTTGGGGTTTTTGTCTGTGGGATATGCCTTACAGACTGAAAATCTATTGTTCCATTTCTCTTCTTC.

## Discussion

**Figure 5:**
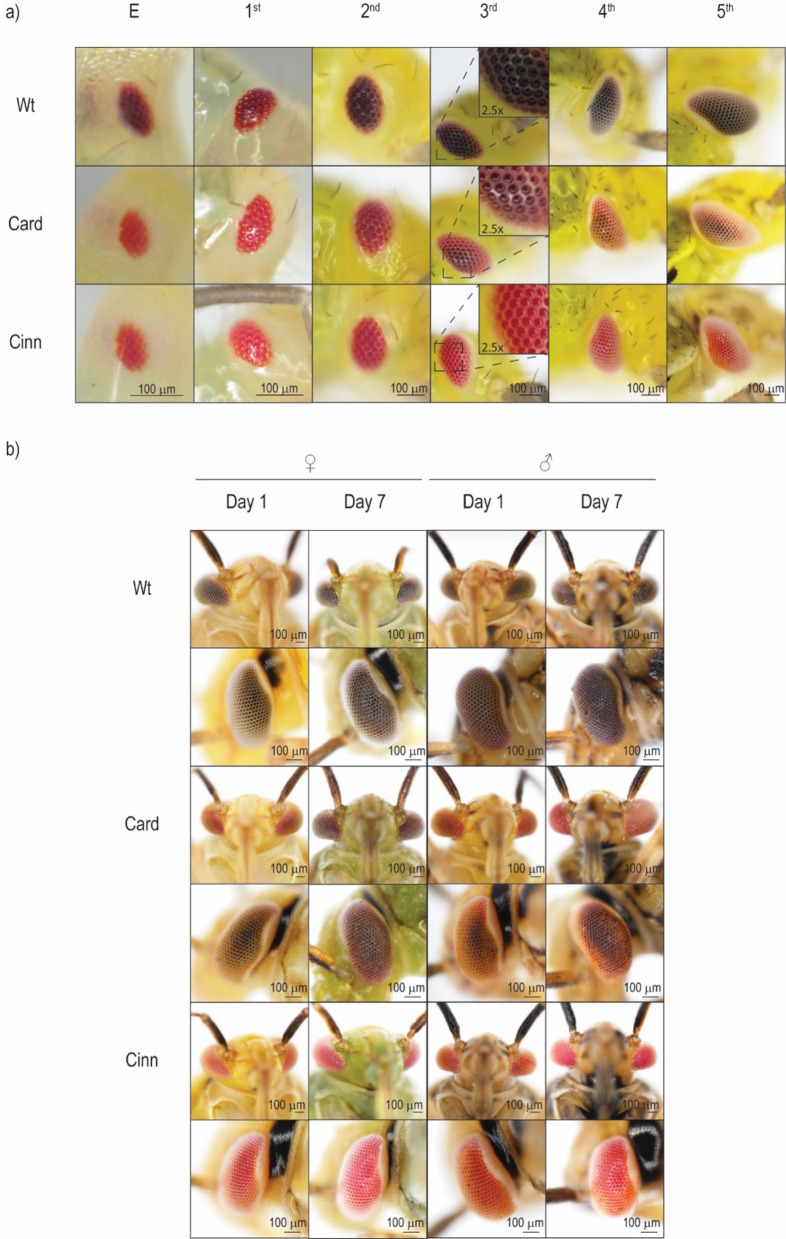
Eye pigmentation phenotypes for the CRISPR/Cas9 *Lygus hesperus* mutant strains. (**a**) Egg (E) and nymphal instars (1st, 2nd, 3rd, 4th, and 5th) corresponding to representative wild-type, Card, and Cinn insects. Insets show 2.5 × magnification of the 3rd instar eye, which underscores gradual reversion to wild-type coloration in the Card line. (**b**) Ventral and lateral views of female and male eyes at 1-day and 7-days post-emergence.

Successful use of CRISPR/Cas9 in *L. hesperus* provides a first step towards the use of contemporary molecular control strategies against this pest species, as well as in other related species. The results here show that CRISPR editing of two *L. hesperus* genes (*LhCd* and *LhCn*) resulted in heritable mutations that affected eye pigmentation across nymphal and adult development. RNAi knockdown of *L. hesperus* genes involved in eye pigment transport in late 5th instars produced adults exhibiting primarily wild-type eyes apart from a red line extending from the rostrum to the antenna along the medial margins^28^. The extent of the change varied by individual and was often difficult to distinguish without close examination. It is evident that eye pigmentation in *L. hesperus* is a continuous process throughout the entire course of development (Fig. 5), which can only be marginally impacted by transient knockdown from RNAi. This attribute led us to attempt CRISPR/Cas9 knockout of the same genes previously targeted to produce a more pronounced and lasting change in phenotype. Our strongest result was with *LhCn*; CRISPR/Cas9-mediated knockout of this gene yielded persistent bright red eyes that were strikingly different from the typical brown coloration in wild-type (Fig. 5). Sequencing of the sgRNA target sites showed multiallelic mutations that confirmed *LhCn* was indeed knocked out. Although the final eye color appears to be species dependent, the bright red eye phenotype in the Cinn strain is consistent with Cinnabar functioning in ommochrome transport/biosynthesis, as has been found in knockdown or knockout studies in *N. lugens*^22^, *Nasonia vitripennis*^33,34^, *Aedes aegypti*^35^, *T. castaneum*^36^, and *B. mori*^37^. It is, however, possible that *LhCn* has other biological roles in *L. hesperus.* The homologous *Cn* gene in *D. melanogaster* modulates post-translational regulation of the mitochondrial fission gene *Drp1*, such that disruption of Cn activity negatively impacts mitochondrial morphology and function^38^. However, we have not observed any changes in *L. hesperus* beyond eye color in this or our previous study of *LhCn*^28^.

Knocking down *cardinal*, another gene in the ommochrome pathway, also impacts eye color in *L. hesperus.* Manipulation of *LhCd* expression in late 5th instars had a moderate impact on eye color^28^, similar to that observed for *N. lugens* in which RNAi yielded eyes with a mixture of red and brown pigment^29^. Unlike the *LhCn* results*, LhCd* knockout mutant eyes appeared to gradually accumulate brown pigment with each successive developmental stage after the 3rd instar (Fig. 5). Although adult eyes in the *LhCd* mutants were redder than that of wild-type individuals, they were substantially darker than *LhCn* mutant eyes. Gradual changes to the eye color have also been found in adults with *crimson* mutations in *Culex pipiens*^39^ and *cardinal* mutants in *Plutella xylostella*^27^. Cardinal functions as the final step in ommochrome biosynthesis to produce xanthommatin. In the absence of a functional *cardinal*, the oxidative 3-hydroxy kynurenine can auto-dimerize to xanthommatin over time^33,40,41^. This may explain the gradual darkening in eye pigmentation that was reminiscent of the wild-type phenotype that we observed in *L. hesperus*.

The *white* gene functions in both pteridine and ommochrome transport^18^, where White dimerizes with either Brown or Scarlet to transport pigments into pigment granules. It is frequently used as a marker of insect genetic manipulation^20,30,42–44^. Although knockdown of *LhW* was frequently fatal in dsRNA-injected 5th instar *L. hesperus* nymphs, it did disrupt pigment accumulation in survivors^28^. Here, knockdown of *LhW* in embryos completely disrupted embryonic development prior to visible eye formation, we were thus unable to confirm if *LhW* is involved in *L. hesperus* eye pigmentation. Similar mortality induced by *white* knockout was observed in *H. armigera*^19^ and *Oncopeltus fasciatus*^30^. Given the pronounced effect of silencing *white*, *LhW* may be essential for embryogenesis in *L. hesperus*, and likely contributes to other key processes. Knockout of *white* in *D. melanogaster* produces retinal degeneration, shortened life span, and progressive loss of ability to climb^45^. The multitude of biological processes affected by *white* indicate that its substrate specificity is not limited to pigment precursors. White has been shown to be expressed in the nervous and excretory system of *Drosophila* and functions in the transport of important substrates like biogenic amines^46^ and cyclic guanosine monophosphate^47^, respectively. The exact functions of *LhW* remain to be elucidated, but are clearly crucial for normal development.

As is evident from the changes to *L. hesperus* eye color, our methods produced efficient and on-target gene editing that was inherited among increasing proportions of progeny for each new generation. Both Card and Cinn colonies have been reared beyond 10 generations with no changes in the mutant eye phenotypes, indicating that respective mutant lines can be stably generated under established laboratory rearing conditions without any observable negative impacts on fitness. Functional genomic studies can now be conducted on key genes, such as those regulating development, reproduction, and insecticide resistance. Use of relatively new molecular approaches, such as Receptor Mediated Ovarian Transduction of Cargo (ReMOT Control) and/or branched amphiphilic peptide capsules (BAPC), can also be applied to facilitate and accelerate functional genomic studies in *L. hesperus*. ReMOT Control uses an ovary specific-ligand to target the RNP cargo to developing oocytes by injecting the abdomens of mature adult females^21,34,48–51^. This technology has potential for generating mutant insects at a higher rate and reduced cost. In contrast, BAPC assisted-delivery of CRISPR/Cas9 into the developing oocyte by injecting the BAPC-RNP mixture near the ovaries may enable enhanced uptake and improve efficiency of editing^48^. The immediate next step in optimizing genetic engineering of *L. hesperus* is to use CRISPR/Cas9 to target genes that reduce fitness, alter sex differentiation, or induce mortality. The generation of conditional gene-drive systems in other model insects exemplifies the feasibility of driving traits with associated negative phenotypes into populations^8,11,52^. Future work in *L. hesperus* will continue to focus on expanding our understanding of basic functional genomics as it relates to pest biology, but also begin to develop new transgenic and/or gene-drive practical approaches for potential pest management purposes.

## Methods

### *Lygus hesperus* rearing

A laboratory colony of *L. hesperus*, collected in Maricopa, AZ, served as the source of insects. Adults were maintained in 0.03 m^3^ screened plastic cages containing shredded paper that were housed in an environmental chamber set at 27 ± 1 °C, 40–60% RH, and a 14:10 (L:D) h photoperiod. Fresh green beans (*Phaseolus vulgaris* L.), an artificial diet pack^53^, and a bottle of water with a wick were used to nourish and hydrate the colony and were replaced as needed.

### Embryonic RNAi

dsRNA targeting *LhCd* (MH806847), *LhCn* (MH806848)*,* and *LhW* (MH806842) was produced as described in Brent and Hull (2019) to a concentration of 1 µg/µl. In brief, ~ 500-bp products were PCR amplified from validated plasmid DNAs harboring *LhCd*, *LhCn*, or *LhW* using T7 promoter containing primers (Supplementary Table S4). PCR products were purified then used as templates for in vitro transcription using a MEGAscript RNAi kit (Thermo Fisher Scientific). dsRNAs corresponding to the fluorescent protein gene *venus*, which was injected as a negative control, were similarly generated.

Gel packs, made of Parafilm M (Pechiney Plastic Packaging, Chicago, IL) and filled with carrageenan (1.25% w/v), were provided to *L. hesperus* as an oviposition substrate for one hour. The gel was removed from the packs and the egg-embedded parafilm sheet retained. The parafilm was stretched to release the eggs, which were then transferred to a moistened filter paper using a wet, fine-tip paintbrush. Eggs were aligned in 4 rows of 10 for a total of 40 eggs per treatment per experiment (*LhCd*, *LhCn*, *LhW*, and *venus*) and then gently covered with a No.1, 24 × 40 mm coverslip coated with permanent linerless double-sided Scotch tape (3 M, Maplewood, MN). The bare side of the coverslip was mounted on a glass slide with double-sided tape that adhered to the corners of the coverslip. The slide was placed on a Leica DMIL scope (Allendale, New Jersey). An IM 300 Microinjector (Narishige International USA, Amityville, NY) with a quartz needle loaded with dsRNA was used to inject the embryos at the posterior pole. Needles were produced by pulling capillary tubes with filament using a P-2000 needle puller (Sutter Instrument, Novato, CA) with the following two-line program: Line 1) Heat = 850, Filament = 5, Velocity = 25, Delay = 128; and line 2) Heat = 700, Filament = 5, Velocity = 50, Delay = 150. Needles were beveled using a Model EG-44 micropipette grinder (Narishige) at a 30° angle and an approximate rotor speed of 1800 rpm or 90% of the maximum speed. Needles were backfilled using a Microloader tip (Eppendorf, Enfield, CT). Following injection, coverslips with eggs were placed in a covered plastic petri dish containing 1% agarose (1 g agarose in 100 ml of distilled water), which was sealed with parafilm and placed in a growth chamber with the same settings as the laboratory colony. Images of eggs were taken 5 days post-injection using a Nikon SMZ18 microscope equipped with a Nikon D5-Ri2 camera (Nikon Instruments Inc., Melville, NY).

To confirm knockdown of targeted transcripts, expression of *LhCd*, *LhCn*, and *LhW* was measured by semi-quantitative RT-PCR, using actin (GDHC01004191) as a loading control. Total RNA was isolated from four replicated groups of three eggs using a Quick-RNA Microprep kit (Zymo Research, Irvine, CA). RNA quality and quantity were assessed using the Take3 module on a Synergy H4 Hybrid Multi-Mode Microplate Reader (Biotek Instruments, Winooski, VT). Total RNA (250 ng) was treated with DNase I (New England Biolabs, Ipswich, MA). cDNAs were generated from 250 ng RNA using a SuperScript III First-Strand Synthesis System (Life Technologies) and custom-made random pentadecamers (Integrated DNA Technologies, San Diego, CA). Fragments (~ 500 bp) of the genes of interest were amplified in a 20 μl reaction volume using SapphireAmp Fast PCR Master Mix (Clontech Laboratories Inc., Mountain View, CA) and primers listed in Supplementary Table S4. PCR conditions consisted of an initial denaturation at 95 °C for 2 min followed by 35 cycles of 95 °C for 20 s, 56 °C for 20 s, 72 °C for 30 s, and a final extension at 72 °C for 5 min. Gel images were obtained using an Azure 200 Gel Imaging Workstation (Azure Biosystems, Dublin, CA) and processed in Adobe Photoshop v21.2.12 (Adobe Systems Inc., San Jose, CA). Independent RNAi experiments were repeated three times.

### Design and synthesis of sgRNAs

sgRNAs were designed using *LhCd* and *LhCn* with a focus on identifying guide sites near the 5′-end of the gene using CRISPOR^54^. Both sets of gene-specific sgRNAs were designed in proximity to one another; LhCd1 and 2 are separated by 8 nucleotides while LhCn1 and 2 are separated by 5 nucleotides. sgRNAs were screened for potential off-target sites by BLASTn of the 20-bp target sequence and the PAM sequence against the *L. hesperus* taxid 30085 database. Potential off-target sites were determined by comparing the BLASTn hit sequences that exactly matched the 3′ end of each sgRNA and the PAM sequence.

Double-stranded gBlock DNA fragments were synthesized by Integrated DNA Technologies (Coralville, Iowa), with each containing a T7 RNA polymerase binding site (5′-TAATACGACTCACTATA-3′), the 20-bp *L. hesperus*-specific target region (Supplementary Table S5), and the 80-bp common stem-loop tracrRNA sequence (5′-GTTTTAGAGCTAGAAATAGCAAGTTAAAATAAGGCTAGTCCGTTATCAACTTGAAAAAGTGGCACCGAGTCGGTGCTTTT-3′). Each gBlock was used as a template for sgRNA synthesis using the HiScribe T7 High Yield RNA synthesis Kit (New England Biolabs). Transcribed sgRNAs were purified using RNAClean XP (Thermo Fisher Scientific) following the manufacturer’s protocol.

### Creation of CRISPR/Cas9 eye pigmentation mutant strains

The experimental design included two independent injection groups; the first injections used a Cas9 protein with a nuclear localization signal (PNA Bio, Newbury Park, CA), whereas the second set used the Alt-R *Streptococcus pyogenes* HiFi Cas9 nuclease V3 (Integrated DNA Technology, Coralville, Iowa). The injection mixture consisted of the RNP complex of Cas9 (300 ng/µl) with two sgRNAs each at 150 ng/µl or a total of 300 ng/µl. Each sgRNA was preincubated with Cas9 at room temperature for 15 min and both solutions of RNP were combined to make the injection mixture. Negative controls include “no injection” and water only. Embryos were prepared and injected as previously described for the RNAi experiments. A total of 80 eggs per treatment (LhCd1 + 2, LhCn1 + 2, non-inject) were injected in the first experiment and the second experiment included 160, 80, 80, and 20 eggs for LhCd1 + 2, LhCn1 + 2, non-inject, and water, respectively.

Six to nine days post-injection, 1st instar nymphs that hatched were collected into a 355 ml mesh lidded paper cup and reared to adulthood under rearing conditions identical to those outlined above. Pairs from each subsequent generation were mated using the crossing schemes shown in Fig. 3. Card cross 1 (× 1) between a mutant male and a mutant female produced 19 G_1_ males and 15 G_1_ females. Card × 2, which also crossed mutants of both sexes, generated 9 G_1_ males and 12 G_1_ females. All G_1_ males from Card × 1 were group-mated with females from Card × 2 and vice versa to generate G_2_. To perpetuate the mutant line, G_2_ with red eyes were selected and transferred to new cages. In addition, Card × 3–5 each consisted of one mutant male and four wild type females. The G_1_ progeny with wild-type eye color from Card × 3–5 were combined and group-mated to generate a mix of wild-type and mutant eye color G_2_ progeny. From the resulting G_2_ progeny, 19 males with the mutant eye phenotype were crossed with 38 G_2_ mutant females from the Card × 1 and × 2 lines. Mutant progeny arising from this group-mating, as well as individuals from the ongoing mutant lines of Card × 1 and × 2 formed the Card colony.

To generate the Cinn colony, two mutant G_0_ females were crossed with two wild-type males to produce G_1_ progeny with wild-type eyes. Of these, three females and four males from Cinn × 1 were backcrossed with three males and 13 females from Cinn × 2, respectively. G_2_ progeny with the mutant eye phenotype were selected to perpetuate the Cinn colony. Mutant colonies were reared in 355 ml paper cups covered with mesh lids with up to 50 individuals. To prevent overcrowding in larger groups of > 50 individuals, 1.89 L paper cups were used^55^. Fresh green beans and sunflower seeds were provided twice a week. Diet and oviposition carrageenan packs were placed into rearing cups one week post-adult emergence.

### Sequencing *LhCd* and *LhCn* from mutant eye pigment strains

Representative insects from CRISPR strains that displayed altered eye pigmentation were collected at G_0_, G_1_, G_2_, and/or G_3_ and stored at -80 °C in RNALater (Invitrogen, Carlsbad, CA). gDNA was extracted using a DNeasy Blood and Tissue kit (Qiagen, Hilden, Germany). Total RNA was extracted using TRI Reagent following the manufacturer’s protocol. Total RNA was treated with DNase I (Thermo Fisher Scientific) and cDNA was synthesized using a SuperScript IV First-Strand Synthesis kit (Invitrogen). *LhCd* and *LhCn* were PCR amplified from gDNA and cDNA using a Phusion High-Fidelity PCR kit (Thermo Fisher Scientific) with gene-specific primer pairs (Supplementary Table S4) and thermocycler conditions of 1 cycle at 98 °C for 30 s; 35 cycles at 98 °C for 5 s, 60 °C for 10 s, 72 °C for 5 s; and 1 cycle at 72 °C for 5 min. PCR products were cloned into pJET1.32/blunt vector (Thermo Fisher Scientific) and transformed into One Shot OmniMAX 2 T1 Chemically Competent *E. coli* (Thermo Fisher Scientific). Multiple clones (n = 4–10) from each transformation reaction were Sanger sequenced (Retrogen Inc., San Diego, CA).

## Supplementary Information

Supplementary Information.
