# Supplementary material for: CRISPR-mediated knockout of cardinal and cinnabar eye pigmentation genes in the western tarnished plant bug
Source: Sci Rep. 2022 Mar 22;12:4917. doi: 10.1038/s41598-022-08908-4 (PMC8943060; doi:10.1038/s41598-022-08908-4)

**SUPPLEMENTARY INFORMATION**

**CRISPR-mediated knockout of *cardinal* and *cinnabar* eye pigmentation genes in the western tarnished plant bug**

Chan C. Heu, Roni J. Gross, Kevin P. Le, Dannialle M. LeRoy, Baochan Fan, J. Joe Hull, Colin S. Brent, Jeffrey A. Fabrick*

USDA ARS, U.S. Arid Land Agricultural Research Center, Maricopa, AZ 85138 USA

*Corresponding author [jeff.fabrick@usda.gov](mailto:jeff.fabrick@usda.gov)

**SUPPLEMENTARY INFORMATION includes:**

**Supplementary Methods**

**Supplementary Tables S1-S5**

**Supplementary Figure S1**

**Supplementary Methods**

**CRISPR/Cas9 gene-editing efficiency in *Lygus* *hesperus*.**  Assuming that the efficiency of germline gene-editing by Cas9 is 100% and recessive inheritance for all induced mutations, we expect G_0_ parents with mosaic eyes to produce 100% G_1_ with mutant eyes; therefore, all G_2_ progeny resulting from G_1_ inbreeding should also have mutant eyes. If the G_0_ parents were outcrossed to wild type, then we expect 0% of the G_1_ progeny to show mutant eyes whereas inbreeding of G_1_ will result in 25% of the G_2_ progeny having mutant eyes. To determine the Cas9 gene-editing efficiency, we randomly sampled from different batches of G_2_ arising from Card x1 and x2 and Cinn x1 and x2. We calculated the percentage of mutant to wild-type eyes, then divided this observed percentage by the expected percentage. We then calculated the average efficiency for both *LhCd* and *LhCn* as the overall average gene-editing rate.

**Supplementary Table S1. BLASTn off-target analysis of *LhCd* and *LhCn* single guide RNAs (sgRNAs).**

| BLASTn against nr database limited to *Lygus hesperus* (taxid 30085) | | | | |  |  |  |  |  |  |
| --- | --- | --- | --- | --- | --- | --- | --- | --- | --- | --- |
| LhCd1 |  |  |  |  |  |  |  |  |  |  |
| query | hit |  |  |  |  |  |  |  |  |  |
| GGCGTGTCTGAGCCTCGTTGNGG | query hit is CGG; GGG, AGG, TGG all align across first 20 nt; identical with CGG | | | | | | | |  |  |
| GGCGTGTCTGAGCCTCGTTGNAG | query hit 21 of 23 through C; no differences with other combination | | | | | | |  |  |  |
|  |  |  |  |  |  |  |  |  |  |  |
| Top 30 hits (excluding the intended hit *Cardinal*) | | |  |  |  |  |  |  |  |  |
|  |  |  |  |  |  |  |  |  |  |  |
| subject accession number | % id | alignment length | mismatches | gap opens | q. start | q. end | s. start | s. end | E value | bit score |
| MH806847.1 | 100 | 7 | 0 | 0 | 1 | 7 | 341 | 335 | 116 | 14.4 |
| MH806847.1 | 100 | 7 | 0 | 0 | 10 | 16 | 685 | 679 | 116 | 14.4 |
| MH806847.1 | 100 | 7 | 0 | 0 | 5 | 11 | 410 | 416 | 116 | 14.4 |
| MH806847.1 | 100 | 7 | 0 | 0 | 17 | 23 | 842 | 848 | 116 | 14.4 |
| KT717331.1 | 100 | 9 | 0 | 0 | 12 | 20 | 1208 | 1200 | 7.4 | 18.3 |
| KT717331.1 | 100 | 7 | 0 | 0 | 14 | 20 | 951 | 945 | 116 | 14.4 |
| KT717331.1 | 100 | 7 | 0 | 0 | 3 | 9 | 1131 | 1125 | 116 | 14.4 |
| JQ639213.1 | 100 | 9 | 0 | 0 | 6 | 14 | 1053 | 1045 | 7.4 | 18.3 |
| JQ639213.1 | 100 | 7 | 0 | 0 | 14 | 20 | 762 | 756 | 116 | 14.4 |
| MH806845.1 | 100 | 9 | 0 | 0 | 4 | 12 | 555 | 563 | 7.4 | 18.3 |
| MH806845.1 | 100 | 8 | 0 | 0 | 10 | 17 | 352 | 345 | 29 | 16.4 |
| MH806845.1 | 100 | 7 | 0 | 0 | 5 | 11 | 2 | 8 | 116 | 14.4 |
| MH806845.1 | 100 | 7 | 0 | 0 | 15 | 21 | 655 | 661 | 116 | 14.4 |
| MH806845.1 | 100 | 7 | 0 | 0 | 3 | 9 | 809 | 815 | 116 | 14.4 |
| KU356754.1 | 100 | 9 | 0 | 0 | 1 | 9 | 2098 | 2106 | 7.4 | 18.3 |
| KU356754.1 | 100 | 8 | 0 | 0 | 5 | 12 | 2680 | 2687 | 29 | 16.4 |
| KU356754.1 | 100 | 7 | 0 | 0 | 14 | 20 | 2290 | 2296 | 116 | 14.4 |
| KX584418.1 | 100 | 9 | 0 | 0 | 10 | 18 | 442 | 450 | 7.4 | 18.3 |
| KX584418.1 | 100 | 7 | 0 | 0 | 12 | 18 | 470 | 464 | 116 | 14.4 |
| KF048098.1 | 100 | 9 | 0 | 0 | 7 | 15 | 172 | 180 | 7.4 | 18.3 |
| KF048098.1 | 100 | 7 | 0 | 0 | 15 | 21 | 572 | 578 | 116 | 14.4 |
| U06476.1 | 100 | 9 | 0 | 0 | 10 | 18 | 725 | 733 | 7.4 | 18.3 |
| U06476.1 | 100 | 8 | 0 | 0 | 8 | 15 | 97 | 90 | 29 | 16.4 |
| U06476.1 | 100 | 7 | 0 | 0 | 13 | 19 | 1697 | 1691 | 116 | 14.4 |
| U06476.1 | 100 | 7 | 0 | 0 | 17 | 23 | 621 | 627 | 116 | 14.4 |
| MN230873.1 | 100 | 8 | 0 | 0 | 11 | 18 | 894 | 887 | 29 | 16.4 |
| MN230873.1 | 100 | 8 | 0 | 0 | 16 | 23 | 1414 | 1407 | 29 | 16.4 |
| MN230873.1 | 100 | 8 | 0 | 0 | 13 | 20 | 3490 | 3483 | 29 | 16.4 |
| MN230873.1 | 100 | 8 | 0 | 0 | 16 | 23 | 2123 | 2130 | 29 | 16.4 |
| MN230873.1 | 100 | 7 | 0 | 0 | 8 | 14 | 446 | 440 | 116 | 14.4 |
| MN230873.1 | 100 | 7 | 0 | 0 | 17 | 23 | 498 | 492 | 116 | 14.4 |
|  |  |  |  |  |  |  |  |  |  |  |
| LhCd2 |  |  |  |  |  |  |  |  |  |  |
| query | hit |  |  |  |  |  |  |  |  |  |
| GAGAGGCTCTGCCTCCAGCANGG | query hit is CGG; GGG, AGG, TGG all align across first 20 nt; identical with CGG | | | | | | | |  |  |
| GAGAGGCTCTGCCTCCAGCANAG | query hit 21 of 23 through C; no differences with other combination | | | | | | |  |  |  |
|  |  |  |  |  |  |  |  |  |  |  |
| Top 30 hits (excluding the intended hit *Cardinal*) | | |  |  |  |  |  |  |  |  |
|  |  |  |  |  |  |  |  |  |  |  |
| subject accession number | % id | alignment length | mismatches | gap opens | q. start | q. end | s. start | s. end | E value | bit score |
| MH806847.1 | 100 | 7 | 0 | 0 | 2 | 8 | 1158 | 1164 | 116 | 14.4 |
| MH806847.1 | 100 | 7 | 0 | 0 | 3 | 9 | 664 | 658 | 116 | 14.4 |
| MH806847.1 | 100 | 7 | 0 | 0 | 7 | 13 | 1108 | 1102 | 116 | 14.4 |
| MH806847.1 | 100 | 7 | 0 | 0 | 13 | 19 | 1195 | 1189 | 116 | 14.4 |
| AF356841.1 | 100 | 10 | 0 | 0 | 8 | 17 | 140 | 131 | 1.9 | 20.3 |
| AF356841.1 | 100 | 8 | 0 | 0 | 7 | 14 | 605 | 612 | 29 | 16.4 |
| AF356841.1 | 100 | 7 | 0 | 0 | 15 | 21 | 718 | 724 | 116 | 14.4 |
| AF356841.1 | 100 | 7 | 0 | 0 | 12 | 18 | 827 | 833 | 116 | 14.4 |
| AF356841.1 | 100 | 7 | 0 | 0 | 10 | 16 | 90 | 84 | 116 | 14.4 |
| AF356841.1 | 100 | 7 | 0 | 0 | 2 | 8 | 301 | 295 | 116 | 14.4 |
| KU356753.1 | 100 | 9 | 0 | 0 | 3 | 11 | 467 | 475 | 7.4 | 18.3 |
| KU356753.1 | 100 | 8 | 0 | 0 | 7 | 14 | 1447 | 1454 | 29 | 16.4 |
| KU356753.1 | 100 | 7 | 0 | 0 | 12 | 18 | 683 | 677 | 116 | 14.4 |
| KU356753.1 | 100 | 7 | 0 | 0 | 12 | 18 | 1421 | 1415 | 116 | 14.4 |
| KT717333.1 | 100 | 9 | 0 | 0 | 12 | 20 | 2915 | 2923 | 7.4 | 18.3 |
| KT717333.1 | 100 | 8 | 0 | 0 | 5 | 12 | 1577 | 1570 | 29 | 16.4 |
| KT717333.1 | 100 | 8 | 0 | 0 | 12 | 19 | 2323 | 2316 | 29 | 16.4 |
| KT717333.1 | 100 | 7 | 0 | 0 | 13 | 19 | 83 | 89 | 116 | 14.4 |
| KT717333.1 | 100 | 7 | 0 | 0 | 13 | 19 | 374 | 380 | 116 | 14.4 |
| KT717333.1 | 100 | 7 | 0 | 0 | 16 | 22 | 927 | 933 | 116 | 14.4 |
| KT717333.1 | 100 | 7 | 0 | 0 | 13 | 19 | 1169 | 1175 | 116 | 14.4 |
| KT717332.1 | 100 | 9 | 0 | 0 | 10 | 18 | 920 | 928 | 7.4 | 18.3 |
| KT717332.1 | 91.67 | 12 | 1 | 0 | 7 | 18 | 287 | 298 | 29 | 16.4 |
| KT717332.1 | 100 | 7 | 0 | 0 | 8 | 14 | 466 | 472 | 116 | 14.4 |
| KF048101.1 | 100 | 9 | 0 | 0 | 6 | 14 | 29 | 37 | 7.4 | 18.3 |
| KF048101.1 | 100 | 8 | 0 | 0 | 11 | 18 | 615 | 608 | 29 | 16.4 |
| KF048101.1 | 100 | 7 | 0 | 0 | 6 | 12 | 150 | 156 | 116 | 14.4 |
| MT210031.1 | 100 | 9 | 0 | 0 | 11 | 19 | 114 | 122 | 7.4 | 18.3 |
| MT210031.1 | 100 | 8 | 0 | 0 | 8 | 15 | 50 | 57 | 29 | 16.4 |
| MT210031.1 | 100 | 8 | 0 | 0 | 11 | 18 | 544 | 551 | 29 | 16.4 |
| MT210031.1 | 100 | 7 | 0 | 0 | 7 | 13 | 55 | 61 | 116 | 14.4 |
|  |  |  |  |  |  |  |  |  |  |  |
| LhCn1 |  |  |  |  |  |  |  |  |  |  |
| query | hit |  |  |  |  |  |  |  |  |  |
| GACAGCGCAGTCCCGATGTGNGG | query hit is CGG; GGG, AGG, TGG all align across first 20 nt; identical with CGG | | | | | | | |  |  |
| GACAGCGCAGTCCCGATGTGNAG | query hit 21 of 23 through C; no differences with other combination | | | | | | |  |  |  |
|  |  |  |  |  |  |  |  |  |  |  |
| Top 30 hits (excluding the intended hit *Cinnabar*) | | | |  |  |  |  |  |  |  |
|  |  |  |  |  |  |  |  |  |  |  |
| subject accession number | % id | alignment length | mismatches | gap opens | q. start | q. end | s. start | s. end | E value | bit score |
| MH806848.1 | 100 | 7 | 0 | 0 | 2 | 8 | 50 | 44 | 116 | 14.4 |
| MT210027.1 | 100 | 10 | 0 | 0 | 1 | 10 | 493 | 502 | 1.9 | 20.3 |
| EU450667.1 | 100 | 10 | 0 | 0 | 9 | 18 | 850 | 859 | 1.9 | 20.3 |
| EU450666.1 | 100 | 10 | 0 | 0 | 9 | 18 | 850 | 859 | 1.9 | 20.3 |
| MN230873.1 | 100 | 9 | 0 | 0 | 15 | 23 | 2098 | 2106 | 7.4 | 18.3 |
| MN230873.1 | 100 | 8 | 0 | 0 | 12 | 19 | 2031 | 2024 | 29 | 16.4 |
| MN230873.1 | 100 | 7 | 0 | 0 | 2 | 8 | 775 | 781 | 116 | 14.4 |
| MN230873.1 | 100 | 7 | 0 | 0 | 11 | 17 | 176 | 170 | 116 | 14.4 |
| MN230873.1 | 100 | 7 | 0 | 0 | 5 | 11 | 1229 | 1223 | 116 | 14.4 |
| MN230873.1 | 100 | 7 | 0 | 0 | 10 | 16 | 2613 | 2607 | 116 | 14.4 |
| KT717333.1 | 100 | 9 | 0 | 0 | 11 | 19 | 1857 | 1865 | 7.4 | 18.3 |
| KT717333.1 | 100 | 8 | 0 | 0 | 11 | 18 | 2718 | 2725 | 29 | 16.4 |
| KT717333.1 | 100 | 7 | 0 | 0 | 3 | 9 | 3142 | 3136 | 116 | 14.4 |
| EU431963.1 | 100 | 9 | 0 | 0 | 10 | 18 | 851 | 859 | 7.4 | 18.3 |
| MH806843.1 | 100 | 9 | 0 | 0 | 12 | 20 | 1406 | 1398 | 7.4 | 18.3 |
| MH806843.1 | 100 | 8 | 0 | 0 | 6 | 13 | 625 | 618 | 29 | 16.4 |
| MH806843.1 | 100 | 7 | 0 | 0 | 16 | 22 | 1489 | 1495 | 116 | 14.4 |
| JF273639.1 | 100 | 9 | 0 | 0 | 11 | 19 | 1117 | 1109 | 7.4 | 18.3 |
| JF273639.1 | 100 | 8 | 0 | 0 | 5 | 12 | 659 | 652 | 29 | 16.4 |
| JF273639.1 | 100 | 7 | 0 | 0 | 10 | 16 | 1275 | 1269 | 116 | 14.4 |
| LC328270.1 | 100 | 8 | 0 | 0 | 6 | 13 | 350 | 357 | 29 | 16.4 |
| LC328270.1 | 100 | 8 | 0 | 0 | 4 | 11 | 361 | 368 | 29 | 16.4 |
| MG027700.1 | 100 | 8 | 0 | 0 | 6 | 13 | 371 | 378 | 29 | 16.4 |
| MG027700.1 | 100 | 8 | 0 | 0 | 4 | 11 | 382 | 389 | 29 | 16.4 |
| KU194349.1 | 100 | 8 | 0 | 0 | 11 | 18 | 207 | 214 | 29 | 16.4 |
| KF048101.1 | 100 | 8 | 0 | 0 | 5 | 12 | 713 | 720 | 29 | 16.4 |
| KF048101.1 | 100 | 7 | 0 | 0 | 13 | 19 | 44 | 38 | 116 | 14.4 |
| KF048097.1 | 100 | 8 | 0 | 0 | 14 | 21 | 532 | 539 | 29 | 16.4 |
| KF048097.1 | 100 | 8 | 0 | 0 | 8 | 15 | 554 | 561 | 29 | 16.4 |
| KF048097.1 | 100 | 8 | 0 | 0 | 16 | 23 | 273 | 266 | 29 | 16.4 |
| KF048097.1 | 100 | 7 | 0 | 0 | 16 | 22 | 424 | 430 | 116 | 14.4 |
|  |  |  |  |  |  |  |  |  |  |  |
| LhCn2 |  |  |  |  |  |  |  |  |  |  |
| query | hit |  |  |  |  |  |  |  |  |  |
| GCATGATCCACGACCCTAAANGG | query hit is GGG; CGG, AGG, TGG all align across first 20 nt; identical with GGG | | | | | | | |  |  |
| GCATGATCCACGACCCTAAANAG | query hit 21 of 23 through C; no differences with other combination | | | | | | |  |  |  |
|  |  |  |  |  |  |  |  |  |  |  |
| Top 30 hits (excluding the intended hit *Cinnabar*) | | | |  |  |  |  |  |  |  |
|  |  |  |  |  |  |  |  |  |  |  |
| subject accession number | % id | alignment length | mismatches | gap opens | q. start | q. end | s. start | s. end | E value | bit score |
| MH806848.1 | 100 | 23 | 0 | 0 | 1 | 23 | 230 | 252 | 3.28E-08 | 46.1 |
| MH806848.1 | 100 | 8 | 0 | 0 | 10 | 17 | 272 | 279 | 29 | 16.4 |
| MH806848.1 | 100 | 7 | 0 | 0 | 12 | 18 | 100 | 106 | 116 | 14.4 |
| MH806848.1 | 100 | 7 | 0 | 0 | 6 | 12 | 447 | 441 | 116 | 14.4 |
| MH806848.1 | 100 | 7 | 0 | 0 | 6 | 12 | 1257 | 1263 | 116 | 14.4 |
| KT818622.1 | 100 | 12 | 0 | 0 | 4 | 15 | 83 | 72 | 0.12 | 24.3 |
| KT818622.1 | 100 | 7 | 0 | 0 | 5 | 11 | 9 | 15 | 116 | 14.4 |
| KT818622.1 | 100 | 7 | 0 | 0 | 9 | 15 | 270 | 264 | 116 | 14.4 |
| KT818622.1 | 100 | 7 | 0 | 0 | 10 | 16 | 617 | 623 | 116 | 14.4 |
| KT818622.1 | 100 | 7 | 0 | 0 | 7 | 13 | 860 | 866 | 116 | 14.4 |
| MH806843.1 | 100 | 10 | 0 | 0 | 7 | 16 | 110 | 101 | 1.9 | 20.3 |
| MH806843.1 | 100 | 9 | 0 | 0 | 9 | 17 | 1215 | 1223 | 7.4 | 18.3 |
| MH806843.1 | 100 | 8 | 0 | 0 | 14 | 21 | 1095 | 1102 | 29 | 16.4 |
| MH806843.1 | 100 | 7 | 0 | 0 | 2 | 8 | 495 | 501 | 116 | 14.4 |
| MH806843.1 | 100 | 7 | 0 | 0 | 5 | 11 | 936 | 930 | 116 | 14.4 |
| MH806846.1 | 100 | 9 | 0 | 0 | 3 | 11 | 997 | 989 | 7.4 | 18.3 |
| MH806846.1 | 100 | 7 | 0 | 0 | 3 | 9 | 343 | 349 | 116 | 14.4 |
| KX950018.1 | 100 | 9 | 0 | 0 | 13 | 21 | 296 | 304 | 7.4 | 18.3 |
| KU194355.1 | 100 | 9 | 0 | 0 | 13 | 21 | 287 | 295 | 7.4 | 18.3 |
| KU194350.1 | 92.31 | 13 | 1 | 0 | 10 | 22 | 320 | 332 | 7.4 | 18.3 |
| KU194350.1 | 100 | 7 | 0 | 0 | 9 | 15 | 69 | 75 | 116 | 14.4 |
| KU194349.1 | 92.31 | 13 | 1 | 0 | 10 | 22 | 329 | 341 | 7.4 | 18.3 |
| KT717331.1 | 100 | 9 | 0 | 0 | 8 | 16 | 693 | 685 | 7.4 | 18.3 |
| KT717331.1 | 100 | 7 | 0 | 0 | 4 | 10 | 735 | 741 | 116 | 14.4 |
| KF679984.1 | 100 | 9 | 0 | 0 | 14 | 22 | 4985 | 4993 | 7.4 | 18.3 |
| KF679984.1 | 100 | 8 | 0 | 0 | 13 | 20 | 691 | 684 | 29 | 16.4 |
| KF679984.1 | 100 | 8 | 0 | 0 | 3 | 10 | 2195 | 2188 | 29 | 16.4 |
| KF679984.1 | 100 | 8 | 0 | 0 | 13 | 20 | 2681 | 2674 | 29 | 16.4 |
| KF679984.1 | 100 | 8 | 0 | 0 | 13 | 20 | 5145 | 5138 | 29 | 16.4 |
| KF679984.1 | 100 | 8 | 0 | 0 | 13 | 20 | 6528 | 6535 | 29 | 16.4 |
| KF679984.1 | 100 | 8 | 0 | 0 | 13 | 20 | 9382 | 9389 | 29 | 16.4 |
| KF679984.1 | 100 | 8 | 0 | 0 | 13 | 20 | 10738 | 10731 | 29 | 16.4 |

**Supplementary Table S2. Statistics of CRISPR/Cas9 targeting *LhCd* or *LhCn*.**

| Experiment # | Treatment | No. Injected Eggs | % Hatch (n) | % Survival to Adults (n) | % G_0_ Adults with Mutant Phenotype (n) |
| --- | --- | --- | --- | --- | --- |
| 1 | no inject | 80 | 100 (80) | 85.0 (68) | 0 (0) |
|  | LhCd1+2 | 80 | 18.8 (15) | 46.7 (7) | 100 (7) |
|  | LhCn1+2 | 80 | 27.5 (22) | 22.7 (5) | 40 (2) |
|  |  |  |  |  |  |
| 2 | no inject | 80 | 86.3 (69) | 58.0 (40) | 0 (0) |
|  | water | 20 | 15 (3) | 100 (3) | 0 (0) |
|  | LhCd1+2 | 160 | 16.9 (27) | 48.1 (13) | 100 (13) |
|  | LhCn1+2 | 80 | 8.8 (7) | 71.4 (5) | 100 (5) |

**Supplementary Table S3. Efficiency of CRISPR/Cas9 gene editing in *Lygus hesperus*.**

| Strain | Sample # from G_2_ | No. Wild-type Sequences | No. Mutant Sequences | Total No. Sequences | % Observed Mutant Sequences | % Expected Mutant Sequences | Efficiency (%) |
| --- | --- | --- | --- | --- | --- | --- | --- |
| Card | 1 | 6 | 33 | 39 | 84.6 | 100 | 84.6 |
|  | 2 | 7 | 43 | 50 | 86.0 | 100 | 86.0 |
|  | 3 | 0 | 35 | 35 | 100.0 | 100 | 100 |
|  | 4 | 3 | 46 | 49 | 93.9 | 100 | 93.9 |
|  |  |  |  |  |  |  |  |
| Cinn | 1 | 112 | 25 | 137 | 18.2 | 25.0 | 73.0 |
|  | 2 | 22 | 6 | 28 | 21.4 | 25.0 | 85.7 |
|  | 3 | 13 | 3 | 16 | 18.8 | 25.0 | 75.0 |
|  |  |  |  |  |  |  |  |
|  |  |  |  |  |  | Avg. | 85.5 |
|  |  |  |  |  |  | S.D. | 9.6 |

**Supplementary Table S4. Nucleotide primers used to amplify, genotype, and/or DNA sequence *LhCd* and *LhCn*.**

| Target | Template | Primer Name | Sequence^a^ |
| --- | --- | --- | --- |
| *LhCd* | gDNA | Cd.485 F | AAACCAGAGCTAAGGTTGAACATATC |
|  |  | Cd.669 R | ACTTGTGATCACTCTTGTAGTTTGC |
| *LhCn* | gDNA | Cn.129.F | TCGGTCTATAAACCTTGCGCT |
|  |  | Cn.258.R | GACAGGGTCGTAAATGATAGGAGAT |
| *LhCd* | cDNA | Lh cardinal 614 F | CCTTCCTCCGTCTGTTGT |
|  |  | Lh cardinal 1147 R | TGCATCCGTCTGTCAAGT |
| *LhCd* | cDNA | Cd538 F | CGCTCTTCCCAGTACCGAAG |
|  |  | Cd853 R | TACTTCCGCAACAGGACACC |
| *LhCn* | cDNA | Lh cn 392 F | GAGTCAATCCAGCAACGG |
|  |  | Lh cn 922 R | CCTGGCCGTAGAATGGAA |
| *LhCn* | cDNA | Cn.91.F | TACCGGCAAGACCCTAGGAA |
|  |  | Cn.412.R | CACCCGTTGCTGGATTGACT |
| *LhW* | cDNA | Lh ABCG10 12 F | TACCGACGAATCTGAGCC |
|  |  | Lh ABCG10 529 R | GAACGCCGTTGATAGAGC |
| *actin* | cDNA | Lh actin 593 F | GCTACTCCTTCACGACCA |
|  |  | Lh actin 1121 R | TTCCTGTGGACGATGGAG |
| *LhCd* | plasmid | Lh cardinal 614 F + T7 | **TAATACGACTCACTATAGGGAGA**CCTTCCTCCGTCTGTTGT |
|  |  | Lh cardinal 1147 R + T7 | **TAATACGACTCACTATAGGGAGA**TGCATCCGTCTGTCAAGT |
| *LhCn* | plasmid | Lh cinnabar 392 F + T7 | **TAATACGACTCACTATAGGGAGA**GAGTCAATCCAGCAACGG |
|  |  | Lh cinnabar 922 R + T7 | **TAATACGACTCACTATAGGGAGA**CCTGGCCGTAGAATGGAA |
| *LhW* | plasmid | Lh white 12 F + T7 | **TAATACGACTCACTATAGGGAGA**TACCGACGAATCTGAGCC |
|  |  | Lh white 529 R + T7 | **TAATACGACTCACTATAGGGAGA**GAACGCCGTTGATAGAGC |
| *venus* | plasmid | Venus + T7 F | **TAATACGACTCACTATAGGGAGA**ATGGTGAGCAAGGGCG |
|  |  | Venus + T7 R | **TAATACGACTCACTATAGGGAGA**GATTAGGCGGCGGTCACGCG |

^a^T7 promoter sequence shown in bold.

**Supplementary Table S5. *LhCd* and *LhCn* Single Guide RNAs (sgRNAs).**

| gRNA name | Guide Sequence (5' to 3')^a^ |
| --- | --- |
| LhCd1 | ACATAAGCTAATACGACTCACTATA**GGCGTGTCTGAGCCTCGTTG**GTTTTAGAGCTAGAAATAGCAAGTTAAAATAAGGCTAGTCCGTTATCAACTTGAAAAAGTGGCACCGAGTCGGTGCTTTT |
| LhCd2 | ACATAAGCTAATACGACTCACTATA**GAGAGGCTCTGCCTCCAGCA**GTTTTAGAGCTAGAAATAGCAAGTTAAAATAAGGCTAGTCCGTTATCAACTTGAAAAAGTGGCACCGAGTCGGTGCTTTT |
| LhCn1 | ACATAAGCTAATACGACTCACTATA**GACAGCGCAGTCCCGATGTG**GTTTTAGAGCTAGAAATAGCAAGTTAAAATAAGGCTAGTCCGTTATCAACTTGAAAAAGTGGCACCGAGTCGGTGCTTTT |
| LhCn2 | ACATAAGCTAATACGACTCACTATA**GCATGATCCACGACCCTAAA**GTTTTAGAGCTAGAAATAGCAAGTTAAAATAAGGCTAGTCCGTTATCAACTTGAAAAAGTGGCACCGAGTCGGTGCTTTT |

^a^Target-specific sequence shown in bold.

**Figure S1**. **Semi-quantitative RT-PCR analysis of *LhCd*, *LhCn*, and *LhW* knockdown.** Uncropped gel images of semi-quantification knockdown shown in Fig. 2b for a) *LhCd*, b) *LhCn*, and c) *LhW* transcripts. cDNA quality control was determined by comparison to actin amplification from each sample.


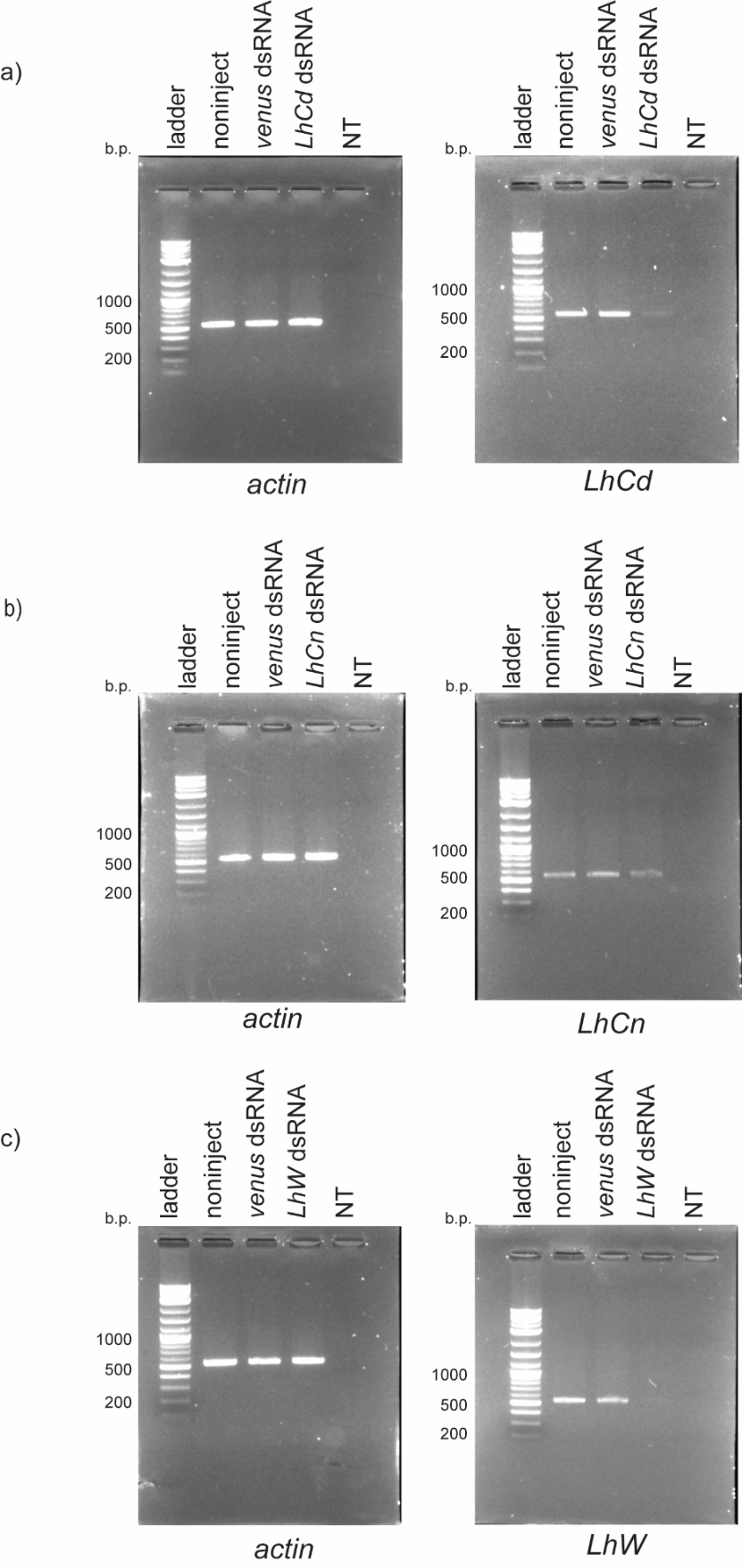

Supplement: Supplementary file 1 — Supplementary Information. [file 41598_2022_8908_MOESM1_ESM.docx]
